# An unusual case of meat allergy

**DOI:** 10.1186/2045-7022-1-S1-P76

**Published:** 2011-08-12

**Authors:** Paola Minale, Elena Penza, Susanna Voltolini, Donatella Bignardi, Paola Dignetti

**Affiliations:** 1 St Martino Hospital, Allergy Unit, Genoa, Italy; 2 ASL22, AL, Italy, # Emergency Dept , Villa Scassi Hospital, Allergy Dept, Genoa, Italy

## 

A 35 years old woman presented with allergic rhinitis and asthma with cat epithelium's sensitivity (Skin prick test positive) treated with antihistamines, nasal and bronchial steroids.

The patient has been maintening the cat in her apartment. After a couple of years from the onset of respiratory allergic disease, she presented an oral allergy syndrome type clinical reaction, gastric pain and diarrhea at once after eating cooked pork meat . Skin prick test (SPT) performed with commercial extracts and fresh meat were positive for pork meat according with the 'pork-chat syndrome'.

Subsequently she presented the same symptoms after eating goat cheese and goat meat.A prick by prick for the culprit foods confermed the food allergy. Recently the same symptoms presented after eating not well cooked beef meat. In her personal history we focused on an anaphylactic reaction occurred at 23 y.o. after injection of antirabbit serum used for antipneumococcal vaccination. The specific IGEs dectection (microarray IMMUNOCAP ISAC system) was positive only for :bovine (Bos d6), cat (Fel d2) ,dog (Can f3), horse (Equ c3) serum albumin and cat, dog lipocalin (Fel d 4, Can f 1).No other foods or respiratory allergens specific IGEs were detected.

Meat allergy is a rare allergic disorder, more frequent during the first year of life but unusual in adults.

Unusually the sensitization to pork meat presented before the goat and beef allergy.

Interestingly no milk allergy was found associated to meat allergy (while in children is frequent ). As it's common the patient presented cat sensitivity and subsequently a crossreactivity towards dog,cat and beef serum albumins. The patient avoids pork, sheep,goat meat but she can eat well cooked beef meat.

